# Leptin-Induced Endothelium-Independent Vasoconstriction in Thoracic Aorta and Pulmonary Artery of Spontaneously Hypertensive Rats: Role of Calcium Channels and Stores

**DOI:** 10.1371/journal.pone.0169205

**Published:** 2017-01-13

**Authors:** Samantha Gomart, Caroline Gaudreau-Ménard, Pascale Jespers, Omer Gurkan Dilek, Emeline Hupkens, Aliénor Hanthazi, Robert Naeije, Christian Melot, Nathalie Labranche, Laurence Dewachter, Kathleen Mc Entee

**Affiliations:** 1 Laboratory of Physiology and Pharmacology, Université Libre de Bruxelles, Campus Erasme, Brussels, Belgium; 2 Faculty of Medicine, University of Montréal, Montréal, Canada; 3 Faculty of Veterinary Medicine, Department of Anatomy, University of Mehmet Akif Ersoy, Burdur, Turkey; 4 Emergency Department, Erasme University Hospital, and Biostatistics Université Libre de Bruxelles, Campus Erasme, Brussels, Belgium; 5 Laboratory of Physiology and Pharmacology, Université Libre de Bruxelles, Campus La Plaine, Brussels, Belgium; The University of Manchester, UNITED KINGDOM

## Abstract

Decreased leptin-induced endothelium-dependent vasodilation has been reported in spontaneously hypertensive rats (SHR). Here, we report leptin-induced vasoconstriction in endothelium-denuded pulmonary artery and thoracic aorta from SHR and sought to characterize calcium handling underlying these mechanisms. Vasoreactivity to leptin was evaluated on pulmonary artery and thoracic aorta rings from 18 weeks old male SHR with or without calcium free medium, caffeine + thapsigargin + carbonyl cyanide-4-trifluoromethoxyphenylhydrazone emptying intracellular calcium stores, nifedipine a voltage-gated calcium channel inhibitor, SKF-96365 a transient receptor potential cation channels (TRPC) inhibitor, wortmaninn, a phosphatidylinositide 3-kinases (PI3K) inhibitor, or PD98059 a mitogen-activated protein kinase kinase (MAPKK) inhibitor. Calcium imaging was performed on cultured vascular smooth muscle cells incubated with leptin in presence or not of wortmaninn or PD98059. Leptin induced vasoconstriction in denuded pulmonary artery and thoracic aorta from SHR. Response was abolished when intra- or extracellular calcium stores were emptied, after blocking TRPC or voltage-dependent calcium channels or when using MAPKK or PI3K inhibitors. In vascular smooth muscle cells, leptin increased intracellular calcium. This rise was higher in SHR and abolished by MAPKK or PI3K inhibitors. TRPC6 gene expression was upregulated in arteries from SHR. Leptin-induced vasoconstriction in denuded arteries of SHR requires intracellular stores and is TRPC- and voltage-gated calcium channels dependent. Intracellular calcium increase is more pronounced in spontaneously hypertensive rats.

## 1. Introduction

Beside its role in food intake and energy homeostasis control, the adipokine leptin may be involved in the regulation of blood pressure. In addition to its ability to stimulate the sympathetic nervous system activity that results in vasoconstriction [1–3] acute leptin administration induces endothelium-dependent vasodilatation through nitric oxide and endothelium-derived hyperpolarizing factor release leading to a neutral effect on blood pressure [4,5]. The leptin-induced vasodilatation observed in vitro is abolished by endothelial denudation, suggesting the essential role of the endothelium [6]. However, it has already been demonstrated that leptin receptor is present on vascular smooth muscle cells and that these cells may represent a direct target for leptin. Leptin indeed reduces angiotensin II-induced vasoconstriction in endothelium denuded arteries [7].

Upregulation of the leptin-leptin receptor axis has been shown to contribute to the pathogenesis of pulmonary arterial hypertension in humans and to experimental hypoxia-induced pulmonary hypertension in rats [8,9]. Moreover, we recently showed that the leptin-induced endothelium-dependent pulmonary arterial relaxation after endothelin-1 precontraction was decreased in spontaneously hypertensive rats (SHR). In this study, the altered response to leptin was attributed to the pulmonary endothelial dysfunction present in SHR [10]. However, already in 1989, Guazzi and associates demonstrated that, in hypertension, the pulmonary and systemic circulations shared joint mechanisms of vasoconstriction directly related to increased calcium in vascular smooth muscle cells [11]. Cytosolic calcium concentration is directly coupled to smooth muscle contraction. Opening of calcium channels in the plasma membrane and in the endoplasmic reticulum leads to passive calcium entry and increased intracellular calcium [12,13]. Enhanced calcium entry via upregulation of TRPC or L-type voltage-dependent calcium channels represent a critical mechanism involved in the development of pulmonary and systemic hypertension [14–16]. Both calcium channels have been shown to be essential in leptin-induced calcium entry in neurons [17,18].

In the present study, we therefore compared the effects of leptin on isometric contraction of endothelium-denuded thoracic and pulmonary artery rings from SHR and control Wistar rats. We evaluated the contribution of plasma membrane calcium channels and of intracellular calcium stores. We studied leptin-induced intracellular calcium concentration variations in vascular smooth muscle cells and intracellular signalling pathways.

## 2. Methods

The present study was approved by the Institutional Animal Care and Use Committee of the Faculty of Medicine of the *Université Libre de Bruxelles* (Brussels, Belgium) and was conducted in accordance with the “Guide for the Care and Use of Laboratory Animals” published by the US National Institutes of Health (NIH publication no. 85–23, revised 1996).

### 2.1. Animals and sample preparation

Experiments were conducted in 18-week old male SHR (Charles River, L’Arbres le Cedex, France) and control Wistar rats (Janvier, Le Genest Saint-Isle, France), weighing respectively 305 ± 4 and 370 ± 26 g. During the acclimatization period, the rats were housed in a temperature (21°C)- and relative humidity (60%)-controlled room with a 12-hour light and dark cycle exposition.

After euthanasia of the animals with carbon dioxide, thoracic aorta and pulmonary artery were carefully excised, cleaned of blood and removed of adhesive fat and connective tissue. After endothelium-denudation, artery sections were placed in Krebs-Henseleit solution (118.1 mmol/L NaCl; 4.7 mmol/L KCl; 1.2 mmol/L MgSO_4_; 1.2 mmol/L KH_2_PO_4_; 2.5 mmol/L CaCl_2_; 25 mmol/L NaHCO_3_; 5.1 mmol/L glucose) for experiments of vasoreactivity or snap-frozen in liquid nitrogen and stored at -80°C for pathobiological evaluation.

### 2.2. Vascular reactivity

Experiments of vasoreactivity were performed as previously described [10]. Briefly, for each animal, two 3 mm-length thoracic aortic and two 3 mm-length pulmonary artery rings were endothelium-denuded by rubbing the intimal surface of the vascular lumen with a surgical steel rod.

Thoracic aortic and pulmonary artery rings were mounted on stainless steel hooks and placed under a resting tension of 1000 mg and 600 mg respectively, in 5 mL-organ baths filled with Krebs-Henseleit solution bubbled with 95% O_2_ and 5% CO_2_ at 37°C. After a 60-min period of equilibration, thoracic aortic and pulmonary artery rings were contracted with KCl (80 mmol/L; Sigma-Aldrich, Bornem, Belgium) to assess their contractility. Subsequently, segments were contracted with phenylephrine hydrochloride (1 μmol/L; Sigma-Aldrich) before the addition of acetylcholine chloride (100 μmol/L; Sigma-Aldrich). The absence of endothelium was confirmed by a vascular tension following acetylcholine treatment exceeding 80% of the phenylephrine-induced pre-contraction. Artery segments that did not match this criterion were excluded from the present study.

After a washout period, allowing the rings to return to a basal vascular tone, rat leptin (Sigma-Aldrich) was tested between 1 pmol/L to 100 nmol/L in thoracic aortic and pulmonary artery segments from SHR and control Wistar rats. The same protocol was repeated in thoracic aortic and pulmonary artery rings from SHR after: (a) a 10-min incubation in calcium free Krebs-Henseleit solution with EGTA (1 μmol/L; Sigma-Aldrich), (b) a 15-min incubation with the L-type voltage dependent calcium blocker nifedipine (3 μmol/L; Sigma-Aldrich), (c) a 15-min incubation with the transient receptor potential cation (TRPC) channel blocker SKF-96365 (30 μmol/L; Sigma-Aldrich), (d) a 25-min pretreatment with caffeine (30 mmol/L; Sigma-Aldrich), thapsigargin (100 nmol/L; Sigma-Aldrich) and carbonyl cyanide-4-trifluoromethoxyphenylhydrazone (FCCP; 100 nmol/L; Sigma-Aldrich) allowing to empty intracellular calcium stores [19], caffeine being re-introduced at 8.5 min and 13.5 min to confirm endoplasmic reticulum depletion [20], (e) a 30-min incubation with the phosphatidylinositol-3-kinase (PI3K) inhibitor wortmannin (10 μmol/L; Sigma-Aldrich) and (f) a 45-min incubation with the mitogen-activated protein kinase kinase (MAPKK) inhibitor PD98059 (50 μmol/L; Sigma-Aldrich). During the experiment, Krebs-Henseleit solution was changed every 20 min and each procedure was preceded by a washout period allowing vessels return to basal vascular tone.

### 2.3. Isolation and primary culture of rat vascular smooth muscle cells

Smooth muscle cells (SMCs) were cultured from thoracic aorta and pulmonary arteries of SHR and Wistar rats, using the explant technique as previously described [21]. Briefly, arteries were dissected under sterile conditions, cut in small fragments and placed in Dulbecco’s modified Eagle’s medium (Gibco, St-Louis, MO, USA) supplemented with 10% foetal bovine serum (FBS), 50 U/mL of penicillin/streptomycin, 50 μM fungizone, 4 mmol/L L-glutamine and 25 mmol/L HEPES (all from Gibco). SMCs were used between passages 3 and 5. To determine the phenotypic characteristics of cultured SMCs, cells were assessed for the expression of alpha-smooth muscle actin and their morphological appearance [21].

### 2.4. Intracellular calcium measurements

SMCs were seeded on coverslips in 500μL of growth medium and allowed to adhere. After medium removal, the cells were rinsed with Ringer buffer (94 mmol/L NaCl; 4.7 mmol/L KCl; 1.2 mmol/L MgSO_4_; 1.9 mmol/L KH_2_PO_4_; 1.7 mmol/L CaCl_2_; 25 mmol/L NaHCO_3_; 5.5 mmol/L glucose) loaded with the fluorescent calcium indicator dye Fluo-4/AM (5 μmol/L diluted in the Ringer solution; Sigma-Aldrich) and incubated for 60 minutes at 37°C in darkness. After washout with the buffer, cells were placed in the perfusion chamber of a Nikon Diaphot 200 inverted microscope (Nikon, Tokyo, Japan) and illuminated with an excitation wavelength of 485nm. The emission signal from the SMCs was collected at 515nm and recorded within a total time course of 1500 seconds by taking one picture every 10 seconds. Data acquisition was performed with Micro-Manager 1.4.16. and Image J 1.48 imaging software was used for data analysis (University of California, San Francisco, USA). Cells were treated with the phosphatidylinositol-3-kinase (PI3K) inhibitor wortmannin (10 μmol/L; Sigma-Aldrich) or the mitogen-activated protein kinase kinase (MAPKK) inhibitor PD98059 (50 μmol/L; Sigma-Aldrich), 3 minutes before or after leptin stimulation (200 nmol/L). At the end of each experiment, ionomycine (2 μmol/L; Sigma-Aldrich) was added for 2 minutes as positive control. All experiments were carried out at room temperature.

For each cell, intracellular calcium rise was quantified as: (Fc-Ff)/F0, where F0 is the mean value of the six last emitted fluorescent light intensities (recorded during 10 minutes) for the selected cell before drug application and Fc is the peak of fluorescence light intensity of the same cell recorded the last minute of drug application. Background autofluorescence (Ff) was measured at the end of each experiment. The average number of cells per condition was 48 ± 12.

### 2.5. Real-Time Quantitative Polymerase Chain Reaction (RTQ-PCR)

Total RNA was extracted from endothelium-denuded thoracic aorta and pulmonary arteries of SHR and Wistar rats by homogenization according to the method of Chomczynski and Sacchi [22] using TRIzol reagent (Invitrogen, Merelbeke, Belgium), and further purified using RNeasy Mini kit (QIAGEN S.A., Hilden, Germany). RNA concentration was determined by spectrophotometry (Nanodrop ND-1000, Isogen life science, De Meern, The Netherlands) and integrity assessed by visual inspection of GelRed (Biotium, Hayward, California, USA)-stained agarose gels. First strand cDNA was synthesized using SuperScript^™^ II Reverse Transcriptase (Invitrogen), according to the manufacturer’s instructions.

For RTQ-PCR, sense and anti-sense primers were designed using Primer3 program for *rattus norvegicus* transient receptor potential cation channel subfamily C members 1, 3, 4 and 6 (*Trpc1*, *Trpc3*, *Trpc4*, *Trpc6*), ryanodine receptor 2 (*Ryr2*), calcium release-activated calcium modulator 1 (*Orai1*), inositol 1,4,5-trisphosphate receptor type 1 (*Itpr1*), calcium voltage-gated channel auxiliary subunitalpha2/delta1 (*Cacna2d1*), stromal interaction molecule 1 (*Stim1*), glyceraldehyde-3-phosphate dehydrogenase (*Gapdh*) and hypoxanthine-guanine phosphoribosyl transferase (*Hprt1*) mRNA sequences amplification (Table 1). To avoid inappropriate amplification of residual genomic DNA, intron-spanning primers were selected when exon sequences were known and a BLAST analysis was run to check if primer pairs were only matching at the sequence of interest. For each primer, PCR conditions were optimized to obtain only the specific product with an efficiency calculated from dilution curves between 95 and 105%. For each sample, amplification reaction (iCycler system, BioRad Laboratories, Nazareth Eke, Belgium) was performed in triplicate using SYBRGreen PCR Master Mix (Quanta Biosciences, Gaithersburg, Maryland), specific primers and diluted template cDNA. Each plate contained negative and positive controls. Melt curves were produced at the end of each plate processing. Relative RNA expression for each transcript of interest was analyzed using the Pfaffl method [23] by normalization with the housekeeping genes, *Gapdh* and *Hprt1*.

**Table 1:** Primers used for real-time quantitative polymerase chain reaction in *rattus norvegicus* thoracic aorta and pulmonary arteries.

Hypoxanthine phosphoribosyltransferase 1 (Hprt1)	Sense	5’–acaggccagactttgttgga– 3’
	Antisense	5’–tccactttcgctgatgacac– 3’
Glyceraldehyde-3-phosphate dehydrogenase human (Gapdh)	Sense	5'—AAGATGGTGAAGGTCGGTGT—3'
	Antisense	5'—ATGAAGGGGTCGTTGATGG—3'
Transient receptor potential cation channel, subfamily C, member 1 (Trpc1)	Sense	5’–GCCAGCCCTTGAGAGAATAG– 3’
	Antisense	5’–CTTCCAACCCTTCATACCACA– 3’
Transient receptor potentialcation channel, subfamily C, member 3 (Trpc3)	Sense	5’–CAAGTGTCTGGTCGTGTTGG– 3’
	Antisense	5’–AGCCTGCTACAAGGTGCAAT– 3’
Transient receptor potential cation channel, subfamily C, member 4 (Trpc4)	Sense	5'–AGGAATCTGGTGAAGCGGTA– 3'
	Antisense	5'–CGAAGCGGAAGCTAGAAATG– 3'
Transient receptor potential cation channel, subfamily C, member 6 (Trpc6)	Sense	5’–TCCGGGGTAATGAAAACAGA– 3’
	Antisense	5’–CATGTATGCTGGTCCTCGATT– 3’
Ryanodine receptor 2 (Ryr2)	Sense	5’–TGTCAAATCAGCACGAATGG– 3’
	Antisense	5’–TGGATAAGTTCAAGCCGTCA– 3’
Calcium release-activated calcium modulator 1 (Orai1)	Sense	5’–ACCATTCTCTAACACCGGGC– 3’
	Antisense	5’–TCATGGGGAAGGGCATAAGG –5’
Inositol 1,4,5-trisphosphate receptor, type 1 (Itpr1)	Sense	5'–AGTTGGCTCGGCATAACAAA– 3'
	Antisense	5'–GCTGTGTGCTTCGCATAGAA– 3'
Calcium channel, voltage dependent, alpha2/delta subunit1 (Cacna2d1)	Sense	5'–GCAGAACTCCAAACAAGATCG–3’
	Antisense	5'–ACTCACGTCCACCAGAATGAG– 3'
Stromal interaction molecule 1 (Stim1)	Sense	5'–GGCCAGAGTCTCAGCCATAG– 3'
	Antisense	5'–CATAGGTCCTCCACGCTGAT– 3'

### 2.6. Statistical analysis

All values are expressed as mean ± standard error of the mean (SEM). Data were analyzed with a two-factor (groups, doses) analysis of variance (ANOVA) for missing data with repeated measurements on doses and interaction, using the algorithm described by Winer BJ [24]. The software was home written by one of us (C.M.) and checked using SAS 9.1.3.5 (Statistical Analysis Software (SAS), Cary, NC). For the calcium measurement and the relative gene expression, a one-way ANOVA was used. The Bonferroni’s correction was applied on p-value as post hoc test. All comparisons were performed at a significance level of p < 0.05, n represents the number of individual data.

## 3. Results

### 3.1. Leptin induced vasoconstriction of endothelium-denuded thoracic aorta and pulmonary artery from spontaneously hypertensive rats (SHR)

**Fig 1:**
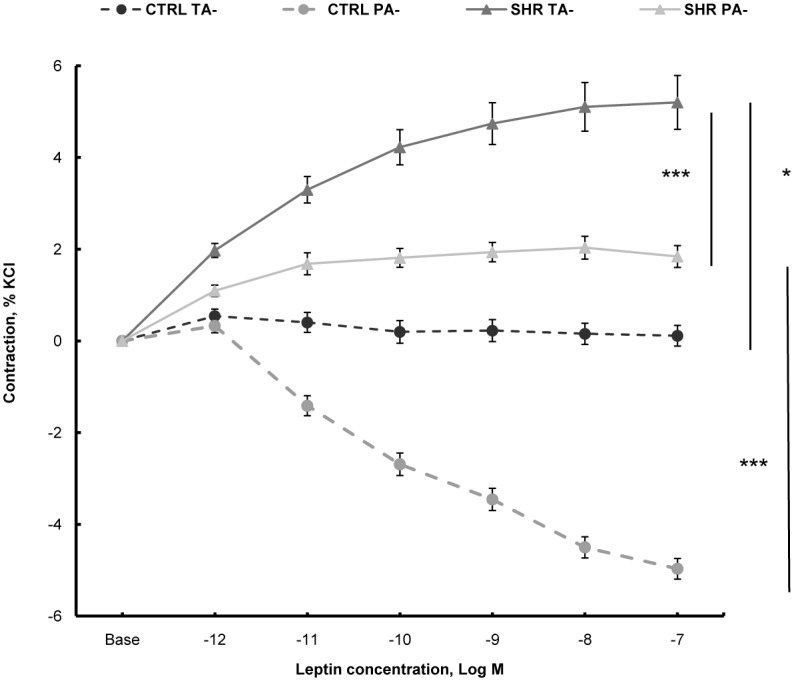
Concentration-response curves to leptin in artery rings from hypertensive rats and normotensive rats. Endothelium-denuded (-) thoracic aortic (TA) and pulmonary artery (PA) rings from spontaneously hypertensive rats (SHR) and control Wistar rats (CTRL) were treated with a cumulative addition of leptin (1 pmol/L to 100 nmol/L). Vasoactive responses were expressed as the percentages of the maximal tension response obtained with 80 mmol/L KCl. Thoracic aortic and pulmonary artery segments were collected in control Wistar rats (n = 8 rings) and in spontaneously hypertensive rats (n = 14 rings). Results are expressed as means ± SEM. ** 0.001<p<0.01, ***p<0.001.

As illustrated in Fig 1, leptin (tested from 1 pmol/L to 100 nmol/L) induced a concentration-dependent contraction of endothelium-denuded pulmonary artery and thoracic aorta from hypertensive rats. The leptin contractile response was more pronounced in thoracic aorta than in pulmonary artery (Fig 1).

In control rats, no response was observed in endothelium-denuded thoracic aorta after leptin treatment, while in endothelium-denuded pulmonary artery, leptin induced a concentration-dependent loss of tone (Fig 1).

### 3.2. Leptin-induced vasoconstriction was dependent on extracellular calcium influx and calcium release from the intracellular store

**Fig 2:**
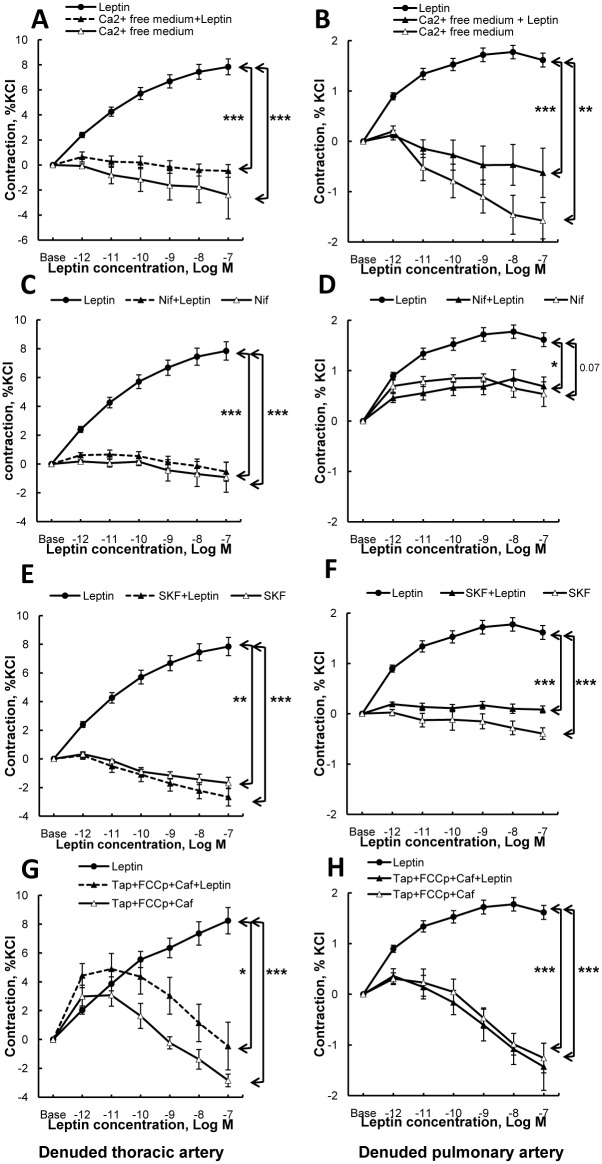
Concentration-response curves to leptin in endothelium-denuded rings from spontaneously hypertensive rats (SHR). Endothelium-denuded thoracic aorta and pulmonary artery rings were treated with a cumulative addition of leptin (1 pmol/L to 100 nmol/L) after preincubation with or without (**A** and **B**) a calcium free medium with EGTA(1 μmol/L); (**C** and **D**) a L-type voltage-dependent calcium channel blocker nifedipine (Nif, 3 μmol/L); (**E** and **F)** a TRPC blocker SKF-96365 (30 μmol/L), or **(G** and **H)** caffeine (Caf) (30 mmol/L) + Thapsigargine (Tap) (100 nmol/L) + FCCP (100 nmol/L) allowing to empty intracellular calcium stores.Vasoactive responses were expressed as the percentages of the maximal tension response obtained with 80 mmol/L KCl. Thoracic aortic and pulmonary artery segments were collected in spontaneously hypertensive rats (n = 20 rings). Results are expressed as means ± SEM. * 0.01<p<0.05, ** 0.001<p<0.01, *** p<0.001.

In endothelium-denuded thoracic aorta and pulmonary artery from SHR, vasoconstriction induced by leptin (1 pmol/L to 100 nmol/L) was totally abolished in presence of a calcium-free medium (Fig 2A and 2B), of a L-type voltage dependent calcium blocker nifedipine (Fig 2C and 2D) or of a TRPC channel blocker SKF-96365(Fig 2E and 2F).

To assess if leptin-induced vasoconstriction in endothelium-denuded pulmonary artery and thoracic aorta from hypertensive rats was dependent on calcium release from intracellular stores, we used a simultaneous treatment of caffeine, thapsigargin and FCCP allowing to empty endoplasmic and mitochondrial intracellular calcium stores before addition of leptin. In these conditions, leptin-induced vasoconstriction was totally suppressed in endothelium-denuded thoracic aorta and pulmonary artery from hypertensive rats (Fig 2G and 2H).

### 3.3. Leptin-induced vasoconstriction was dependent on the MAPKK- and PI3K-pathways

**Fig 3:**
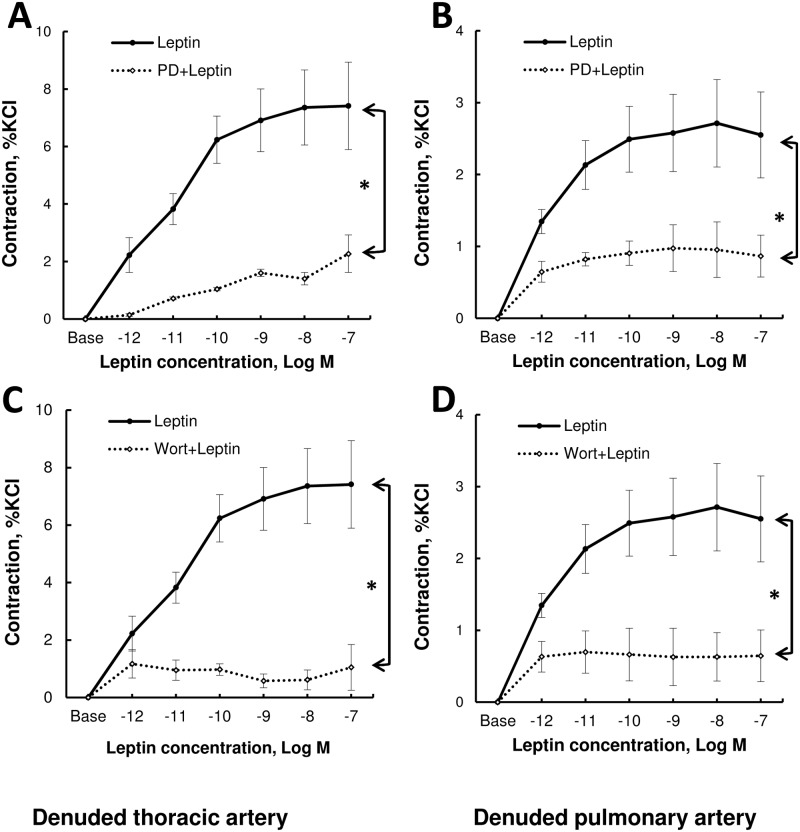
Concentration-response curves to leptin in endothelium-denuded rings from spontaneously hypertensive rats (SHR). Endothelium-denuded thoracic aorta and pulmonary artery rings were treated with a cumulative addition of leptin (1 pmol/L to 100 nmol/L) after preincubation with or without a MAPKK inhibitor PD98059 (PD; 50 μmol/L) (**A** and **B**) or a PI3K inhibitor wortmaninn (wort; 10 μmol/L) (**C** and **D**).Vasoactive responses were expressed as the percentages of the maximal tension response obtained with 80 mmol/L KCl. Thoracic aortic and pulmonary artery segments were collected in spontaneously hypertensive rats (n = 6 rings). Results are expressed as means ± SEM. * 0.01<p<0.05, ** 0.001<p<0.01, *** p<0.001.

Pretreatment with PD98059 or wortmaninn abolished leptin-induced contraction in thoracic aorta (Fig 3A–3C) and pulmonary artery (Fig 3B–3D).

### 3.4. Leptin-induced cytosolic calcium rise was enhanced in hypertensive rats

**Fig 4:**
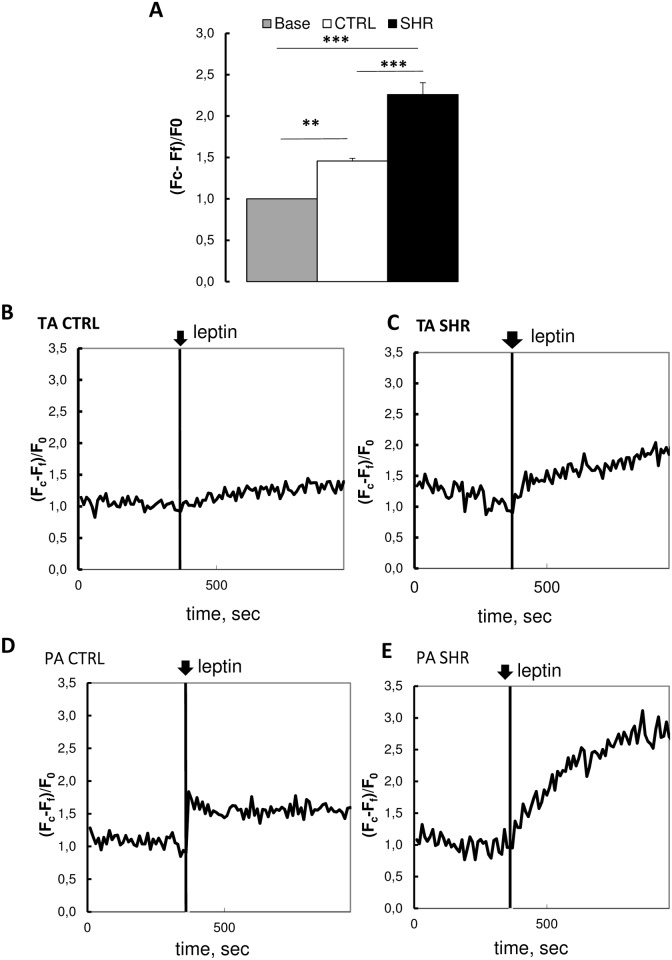
Measurement of cytosolic calcium concentration after leptin treatment in primary vascular smooth muscle cells. (**A**) Comparison of cytosolic calcium concentration after leptin treatment (200 nmol/L) in primary vascular smooth muscle cells [including thoracic aorta (TA) and pulmonary artery (PA) smooth muscle cells]] from control Wistar (CTRL) and spontaneously hypertensive rats (SHR) (n = 48 ± 12 cells). Six control Wistar rats and three SHR were used for primary culture of vascular smooth muscle cells isolated by the explant technique from thoracic aorta and pulmonary artery. Cytosolic calcium concentration was measured as the intracellular calcium fluorescent signals, assessed by (Fc-Ff)/F0, where Fc corresponds to maximal cytosolic fluorescence during the last minute before the addition of the next drug, Ff corresponds to background fluorescence and F0 corresponds to mean fluorescence of the 6 last measurements before the addition of the first drug. The base corresponds to mean fluorescence of the 6 last fluorescent measurements before the addition of the first drug. Results are expressed as means ± SEM.** 0.001**<**p<0.01, ***p<0.001.Time course of the intracellular calcium fluorescent signal (assessed by fluorescence microscopy after loading with the Fluo-4 sensor) after leptin treatment (200 nmol/L) in smooth muscle cells from (B) control thoracic aorta, (C) SHR thoracic aorta, (D) control pulmonary artery, (E) SHR pulmonary artery.

As shown in Fig 4, leptin (200 nmol/L) induced a marked increase in intracellular calcium concentration (assessed by the quantification of Fluo-4 fluorescence intensity). In control rats, SMCs isolated from pulmonary artery and thoracic aorta had an intracellular calcium concentration that rose quickly to a peak corresponding to a 1.5 fold increase in fluorescence compared to baseline, followed by a plateau.

The increase in intracellular calcium concentration induced by leptin was enhanced in vascular SMCs (including those from thoracic aorta and pulmonary artery) by 60% in cells from SHR compared to controls (Fig 4).

### 3.5. Leptin increased intracellular calcium concentration in smooth muscle cells in MAPKK- and PI3K-dependent mechanisms

**Fig 5:**
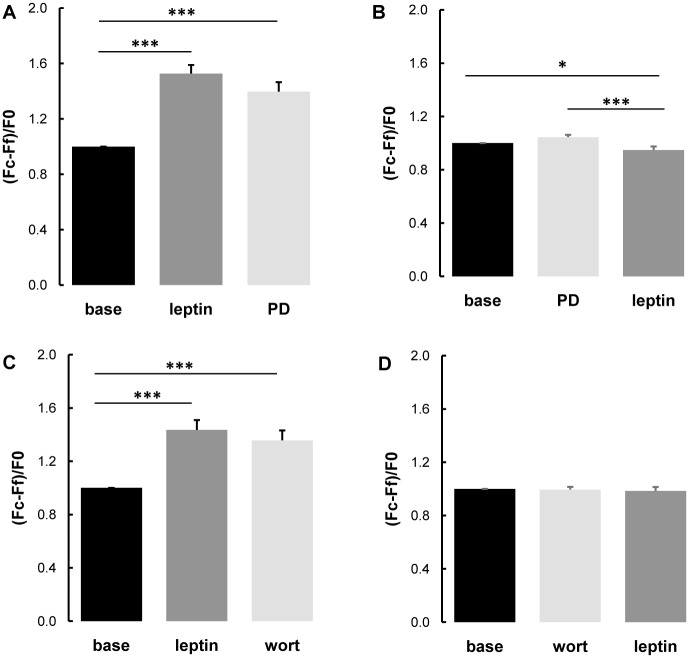
Role of the MAPKK and PI3K signaling in leptin induced response in thoracic aorta cells. Measurements of the intracellular calcium fluorescent signals [assessed by (Fc-Ff)/F0, where Fc corresponds to maximal cytosolic fluorescence during the last minute before the addition of the next drug, Ff corresponds to background fluorescence and F0 corresponds to mean fluorescence of the 6 last measurements before the addition of the first drug] in control thoracic aorta smooth muscle cells treated with (**A**) leptin (200 nmol/L) followed by the addition of a MAPKK inhibitor PD98059 (PD; 50 μmol/L; n = 35 cells), (**B**) MAPKK inhibitor PD98059 (PD; 50 μmol/L) followed by treatment with leptin (200 nmol/L; n = 33 cells), (**C**) leptin (200 nmol/L) followed by the addition of a PI3K inhibitor wortmaninn (wort; 10 μmol/L; n = 31 cells), and (**D**) PI3K inhibitor wortmaninn (wort; 10μmol/L) followed by treatment with leptin (200 nmol/L; n = 30 cells). Six rats were used for primary culture of vascular smooth muscle cells isolated by the explant technique from thoracic aorta and pulmonary artery. Results are expressed as means ± SEM. * 0.01<p<0.05,*** p<0.001.

**Fig 6:**
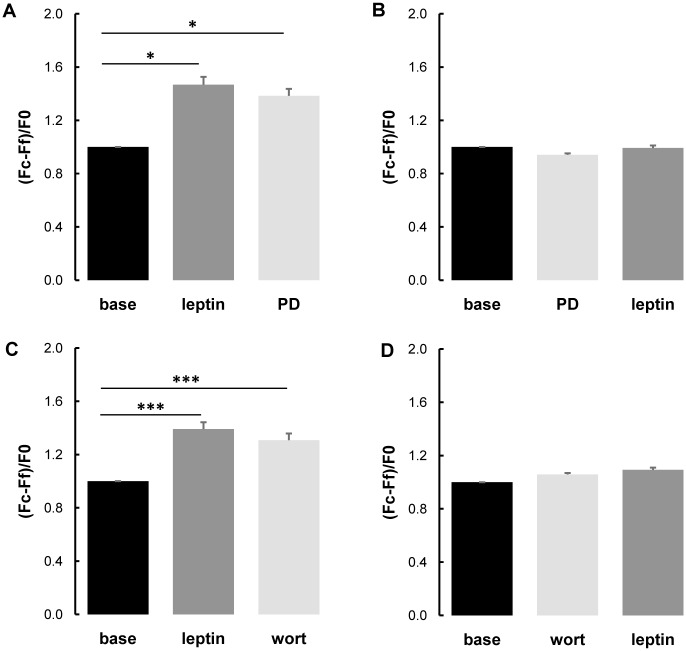
Role of the MAPKK and PI3K signaling in leptin induced response in pulmonary artery cells. Measurements of the intracellular calcium fluorescent signals [assessed by (Fc-Ff)/F0, where Fc means maximal cytosolic fluorescence during the last minute before the addition of the next drug, Ff means background fluorescence and F0 means mean fluorescence of the 6 last measurements before the addition of the first drug] in control pulmonary artery smooth muscle cells treated with (**A**) leptin (200 nmol/L) followed by the addition of a MAPKK inhibitor PD98059 (PD; 50 μmol/L; n = 36 cells), (**B**) MAPKK inhibitor PD98059 (PD; 50 μmol/L) followed by treatment with leptin (200 nmol/L; n = 34 cells), (**C**) leptin (200 nmol/L) followed by the addition of a PI3K inhibitor wortmaninn (wort; 10 μmol/L; n = 34 cells) and (**D**) PI3K inhibitor wortmaninn (wort; 10μmol/L) followed by treatment with leptin (200 nmol/L; n = 31 cells). Six rats were used for primary culture of vascular smooth muscle cells isolated by the explant technique from thoracic aorta and pulmonary artery. Results are expressed as means ± SEM.* 0.01<p<0.05,*** p<0.001.

Treatment with the MAPKK inhibitor PD98059 or the PI3K inhibitor wortmaninn after leptin did not change the intracellular calcium concentration in control thoracic aorta (Fig 5A and 5C) and pulmonary artery (Fig 6A and 6C) SMCs. However, pretreatment with PD98059 or wortmaninn totally prevented leptin-induced increased intracellular calcium concentration in control thoracic aorta (Fig 5B and 5D) and pulmonary artery SMCs (Fig 6B and 6D).

### 3.6. Thoracic aorta and pulmonary artery expression of plasma membrane and reticulum endoplasmic calcium channels and related molecules

**Fig 7:**
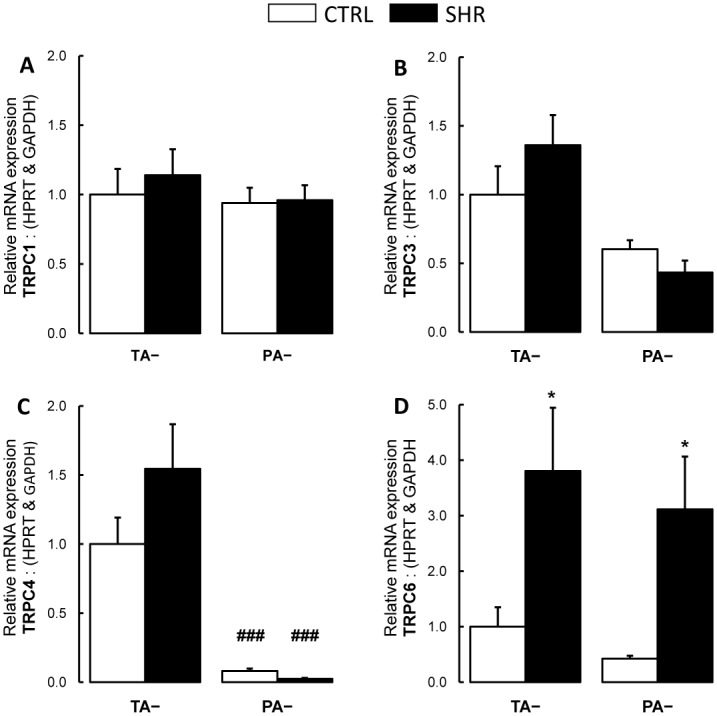
Expression of transient receptor potential cation channels subfamily C (Trpc) members. Relative gene expressions of (**A**) *Trpc1*, (**B**) *Trpc3*, (**C**) *Trpc4* and (**D**) *Trpc6* in pulmonary artery and thoracic aorta from control Wistar (CTRL; n TA = 14 rings; n PA = 14 rings; white bars) and spontaneously hypertensive rats (SHR; n TA = 12 rings; n PA = 16 rings; black bars). Results are expressed as means ± SEM. * 0.01<p<0.05, compared to the corresponding artery from the control group; ### p<0.001 compared to the thoracic artery in the same strain of rats.

**Fig 8:**
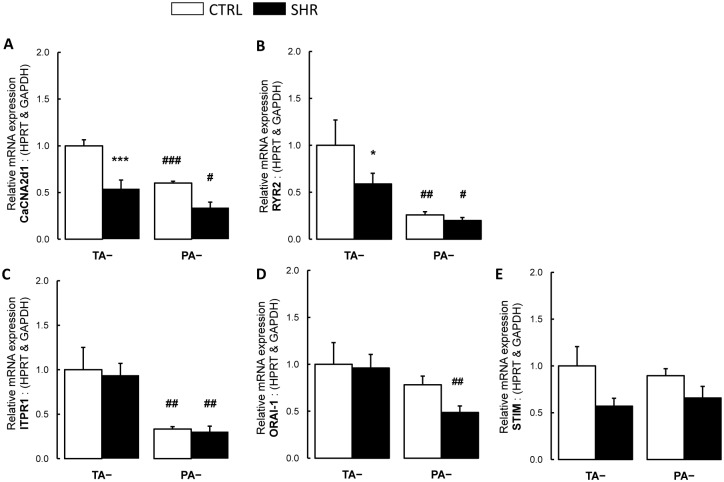
Expression of calcium channels and related molecules. Relative gene expressions of (**A**) the calcium voltage-gated channel auxiliary subunit alpha2/delta1 (*Cacna2d1*); (**B**) the ryanodine receptor 2 (*Ryr2*); (**C**) the inositol 1,4,5-trisphosphate receptor type 1 (*Itrp1*); (**D**) the calcium release-activated calcium modulator-1 (Orai-1) and (**E**) the stromal interaction molecule 1 (*Stim1*) in pulmonary artery and thoracic aorta from control Wistar (CTRL; n TA = 14 rings; n PA = 14 rings; white bars) and spontaneously hypertensive rats (SHR; n TA = 12 rings; n PA = 16 rings; black bars). Results are expressed as means ± SEM. * 0.01<p<0.05, *** p<0.001 compared to the corresponding artery from the control group; # 0.01<p<0.05, ## 0.001<p<0.01, ### p<0.001 compared to the thoracic artery in the same strain of rats.

In control rats, relative gene expressions of *Trpc4* (Fig 7C), L-type voltage dependent calcium channel (*Cacna2d1*; Fig 8A), ryanodine receptor (*Ryr2*; Fig 8B) were lower in pulmonary artery compared to thoracic aorta, while expressions of *Trpc1*, *Trpc3* and *Trpc6* were similar (Fig 7A, 7B and 7D). In hypertensive rats, relative *Trpc6* expression increased in thoracic aorta and pulmonary artery (Fig 7D) compared to controls. Relative gene expressions of *Cacna2d1* and *Ryr2* were decreased in thoracic aorta from SHR (Fig 8A and 8B) compared to controls.

We also evaluated the expression of an intracellular receptor for IP_3_, the *Itrp1*, which mediates calcium release from the endoplasmic reticulum upon stimulation by IP_3_. In both spontaneously hypertensive and control rats, relative gene expression of *Itrp1* was lower in pulmonary artery compared to thoracic aorta (Fig 8C). Expressions of *Itrp1* were similar in hypertensive and control rats (Fig 8C).

*Orai-1* is a membrane calcium channel subunit that is activated by the calcium sensor *Stim1* when calcium stores are depleted. In spontaneously hypertensive rats, relative gene expression of *Orai-1* was less abundant in pulmonary artery compared to thoracic aorta (Fig 8D). In both hypertensive and control rats, *Stim* expression tended to decrease, but did not reach significance (Fig 8E).

## 4. Discussion

The present study shows that leptin induces vasoconstriction in endothelium-denuded thoracic aorta and pulmonary artery from SHR through the opening of *Trpc*, voltage-gated calcium channels and the mobilization of intracellular calcium stores. This is associated with a rise in smooth muscle intracellular calcium in MAP kinase kinase- and PI3 kinase-dependent manners. These pathways participate in leptin-induced vasoconstriction.

Chronic hyperleptinemia has been related to hypertension in rats [25] and in humans [26–29]. Indeed, it has been shown that leptin induces vasoconstriction through the activation of the sympathetic nervous system [30–32]. Leptin also has a vasodilating endothelial-dependent effect involving the release of nitric oxide [6]. Leptin inhibits angiotensin II-induced cytosolic calcium increase and thus vasoconstriction in smooth muscle cells isolated from the aorta of normotensive rats [7]. This effect is blunted in cells from a spontaneously hypertensive strain of rats [33].

In the present study, we found that leptin induced vasoconstriction in endothelium-denuded thoracic aorta and in a lesser extent in pulmonary artery from SHR, while it did not in arteries from control Wistar rats. This suggests that leptin could play a role in modulating vasomotion directly through leptin receptor present on smooth muscle cells [34,35] and therefore contribute to the progression of systemic and pulmonary hypertension in SHR. In contrast, in control normotensive rats, leptin induced a loss of tone in endothelium-denuded pulmonary artery. This illustrates that leptin has variable effects depending on the context.

Hypertension is associated with vasoconstriction due to profound alteration in smooth muscle calcium homeostasis, characterized by increased intracellular calcium concentration. This intracellular increment of calcium results from both calcium influx through voltage-dependent and non-voltage gated calcium channels and calcium release from intracellular stores [36–39]. Here, we found that leptin-induced vasoconstriction was dependent on both voltage-dependent calcium and TRPC channels in thoracic aorta and pulmonary artery from SHR. This is consistent with previous studies showing that calcium influx, particularly in smooth muscle cells from SHR, resulted from the interdependence between these two different types of channels [14, 40]. In addition to a potential direct contribution to calcium influx, *Trpc* channels are thought to promote calcium entry by providing a depolarizing stimulus (sodium and calcium currents) for voltage-dependent calcium channel activation and subsequent smooth muscle cell contraction [41, 42]. Moreover, both L-type voltage-gated calcium and *Trpc* channels have been shown to be essential in leptin-induced calcium entry in neurons [17, 18]. In the present study, leptin-induced vasoconstriction was also suppressed when endoplasmic and mitochondrial calcium stores were emptied. Taken together, these results suggest a tight coupling between plasma membrane and sarcoplasmic calcium handling proteins in mediating smooth muscle contraction induced by leptin.

We also found that leptin induced a rise in intracellular calcium concentration in cultured thoracic aorta and pulmonary artery smooth muscle cells and that this effect was of higher magnitude in cells from SHR. This suggests that leptin stimulates calcium influx from the extracellular compartment and promotes the release of calcium from the intracellular endoplasmic reticulum calcium stores. The former event may occur in association with voltage-sensitive calcium and *Trpc* channels and the latter via the phosphoinositide turnover. This leads to a rise in intracellular calcium concentration, inducing vasoconstriction. The resting cell fluorescence, which is correlated to intracellular calcium concentration, was similar in smooth muscle cells from hypertensive and normotensive rats. This suggests increased calcium recruitment from extra- and intracellular compartments by leptin rather than basal resting differences. This could be explained by the upregulation of receptor- and store-operated channels in smooth muscle cells from SHR, contributing to the vasoconstriction induced by leptin. In spontaneously hypertensive rats, serum leptin concentration is ≈ 3.1 ng/mL (corresponding to 0.18 nmol/L), while it is ≈ 2.5 ng/mL (or 0.15 nmol/L) in normotensive Wistar rats [33]. Cytosolic calcium rise induced by leptin was observed at supraphysiological concentrations (200 nmol/L) while in a previous study, leptin at a physiological concentration (10 nmol/L) did not increase intracellular calcium in aortic SMCs from normotensive Wistar rats [7] and SHR [33]. This suggests that leptin could promote cytosolic calcium rise in pathological conditions characterized by hyperleptinemia, such as obesity or hypertension but not in the physiological state.

To investigate calcium-related signaling pathways in smooth muscle cells, we blocked PI3 kinase and MAPKK signaling. In thoracic aorta and pulmonary artery smooth muscle cells, leptin increased cytosolic calcium concentration in MAPKK- and PI3K-dependent manners. To investigate potential link between pathways implicated in cytosolic calcium rise and vasoconstriction, we tested vasoreactivity to leptin in pulmonary artery and thoracic aorta pre-incubated with PI3K or MAPKK inhibitor. We found that leptin-induced vasoconstriction was dependent on the MAPKK- and PI3K-pathways. Taken together, these results suggest that leptin-induced calcium rise observed at cellular level may lead to vasoconstriction.

Under physiological conditions, leptin reportedly stimulates the expression and activity of inducible nitric oxide synthase (*iNos*) through mechanisms involving JAK2/STAT3 and PI3K/Akt [43]. This increase in NO bioavailability is required for the inhibitory effect of leptin on angiotensin II-induced calcium increase in aortic SMCs and vasoconstriction. However, leptin can also induce the formation of reactive oxygen species (ROS) through the activation of NADPH oxidase enzyme in arterial SMCs [44]. Interestingly, aortic SMCs from SHR showed an impaired leptin-induced NOS activity and NO production as well as an increased basal expression of NADPH oxidase subunit NOX2 [45]. Therefore, in pathological conditions, the ability of leptin to induce NO production might be overridden by the increased ROS formation leading to an increase in cytosolic calcium in SMCs and vasoconstriction.

The importance of the PI3K pathway has already been demonstrated for leptin-induced depolarization of neurons [17,18] and for smooth muscle cell proliferation and migration [35,46]. Indeed, in hypothalamic neurons, leptin-receptor binding activates the janus kinase 2-PI3K pathway. PI3K induces calcium entry by voltage dependent calcium channels and activates phospholipase C to produce IP_3_-induced calcium release from intracellular stores and subsequently store operated calcium entry by *Trpc* [17,18].

Alteration in calcium channel function and/or increase in calcium channel density regulate vasoactive characteristics. Here, we found increased expression of *Trpc6* in thoracic aorta and pulmonary artery from SHR. Expressed by smooth muscle cells, *Trpc6* has been shown to be a major constituent of agonist-induced receptor-operated calcium entry in systemic and pulmonary artery smooth muscle cells [47–49]. Enhanced expression of *Trpcs*, including *Trpc6*, plays a critical role in the increase of pulmonary vascular tone in experimental model of pulmonary hypertension induced by chronic hypoxia exposure [49, 50].

In the present study, expression of L-type voltage-dependent calcium channel was reduced in thoracic aorta and pulmonary artery from SHR. This is in contrast with previous studies showing increased expression of L-type calcium channels in different arteries from SHR [14]. However, House et al. showed that the switch of smooth muscle cell phenotype observed in many vascular diseases from a quiescent to a proliferative motile phenotype is associated with increased expression of TRPC channels and a loss of L-type calcium channels [51].

## 5. Conclusion

In conclusion, the present study shows that, in endothelium-denuded thoracic aorta and pulmonary artery from SHR, leptin induces vasoconstriction in a PI3K and MAPKK manner, by increasing intracellular calcium via voltage dependent and *Trpc* plasma membrane channels and release of intracellular stores. Leptin-induced enhanced intracellular calcium concentration could contribute to the development of hypertension, through altering vasomotion and contributing to vascular remodelling.
